# Optimization of Extraction and Purification of Flavonoids from Stigmaless Floral Residues of *Crocus sativus* L. and Their Stimulatory Effect on Glucose Uptake In Vitro

**DOI:** 10.3390/molecules29143271

**Published:** 2024-07-10

**Authors:** Sunce Chen, Quanhe Lv, Chunhui Liu, Hongxia Yuan, Chunfei Li, Yifan Liu, Wen Zhang

**Affiliations:** 1 School of Life Sciences, East China Normal University, 500 Dongchuan Road, Shanghai 200241, China; 13283059979@163.com (S.C.); 51251300007@stu.ecnu.edu.cn (Q.L.); 15247656365@163.com (H.Y.); 51211300008@stu.ecnu.edu.cn (C.L.); liuyifan19970414@163.com (Y.L.); 2 China National Institute of Standardization, 4 Zhichun Road, Beijing 100191, China; 3 Wenzhou Student Practical School, 1111 Fuzhou Road, Wenzhou 325000, China

**Keywords:** stigmaless floral residues of *Crocus sativus* L., extraction, purification, flavonoids, glucose uptake

## Abstract

Saffron, the dried stigma of *Crocus sativus* L., is a renowned spice and medicinal herb. During its production, a significant amount of floral residues, rich in bioactive compounds, are discarded as agricultural by-products. This study presents a novel approach to the sustainable utilization of these stigmaless floral residues (FRC) by optimizing the extraction and purification of their flavonoids, analyzing their chemical composition, and evaluating their effect on glucose uptake. The extraction of flavonoids from FRC was optimized using single-factor experiments and response surface methodology. The optimal conditions for extraction were an ethanol concentration of 67.7%, a temperature of 67.6 °C, a solid-to-liquid ratio of 1:30, an extraction time of 3 h, and two extractions. The crude extract obtained was then purified using macroporous resin HPD100, selected after comparing the adsorption and desorption characteristics of six different resins. The optimal purification parameters were an adsorption concentration of 40 mg/mL, a loading volume of 7 bed volumes (BV) at a flow rate of 3 BV/h, and 80% ethanol as the eluent with a volume of 4 BV. The resulting flavonoid-enriched extract (FFRC) had an experimental yield of 8.67% ± 0.01 and a flavonoid content of 128.30 ± 4.64 mg/g. The main flavonoids in FFRC were identified as kaempferol glycosides, isorhamnetin glycosides, and quercetin glycosides. Moreover, FFRC significantly stimulated glucose consumption and uptake in C2C12 myotubes, suggesting its potential utility as a natural hypoglycemic agent. This study contributes to the sustainable and value-added utilization of agricultural resources by providing data for the exploitation and application of flavonoids from saffron by-products.

## 1. Introduction

*Crocus sativus* L., also known as saffron crocus, is a perennial herb in the Iridaceae family. It is mainly cultivated in Iran, China, Spain, Morocco, Italy, Greece, and India [1,2]. The stigmas of this plant, commonly called saffron, are valuable spices that are used as flavoring and coloring agents worldwide [3]. They also have a long history of medicinal use dating back to over 3600 years ago [4]. In China, saffron is a traditional Chinese medicine that has been shown to have effects such as promoting blood circulation and removing blood stasis, cooling and detoxifying blood, and alleviating depression [2]. It is mainly used to treat conditions such as irregular menstruation, postpartum thrombosis, and bruises [2]. Pharmacological studies have revealed that saffron has various therapeutic activities, such as antioxidant [5,6], antimicrobial [6], anti-inflammatory [5], anticancer [5,7], and antidepressant [4] effects.

Despite these benefits, saffron production is labor-intensive, requiring careful separation of the stigmas from the other floral parts [5,8]. Each flower has a style that divides into three brilliant red stigmas, each 25–30 mm long and weighing approximately 2 mg [4,9]. To produce 1 kg of dried stigmas, about 300,000 flowers need to be carefully picked [10]. As a result, large quantities of stigmaless floral residues of *C. sativus* (FRC), mainly tepals and stamens [8], constitute about 90% of the total fresh weight and are usually discarded as useless by-products after harvest [5]. In China alone, the annual saffron output is a few tons, which means that tens of tons of FRC are wasted every year, causing a significant loss of resources and severe environmental pollution [11].

However, these by-products have significant economic and pharmacological potential, as they contain various bioactive compounds, such as phenolic compounds [10], flavonoids [8,10], anthocyanins [10], and crocins [8]. These compounds have been shown to have antioxidant [5,12,13], antimicrobial [13], anti-inflammatory [5], antidepressant [10], antiproliferative [14], and hypoglycemic [15] effects. Moreover, among the by-products, the petals have higher phenolic and flavonoid content than stamens and styles [8,10], while stamens have higher crocin content than petals [8]. Crocins are responsible for the color and aroma of saffron and also have antioxidant activity [8]. However, most of the research and utilization of saffron by-products in recent years have focused on petals, which need to be separated from stamens by manual work. This is time-consuming, laborious, impractical, and wasteful. Further, the stamen is another important by-product that contains valuable compounds such as flavonoid glycosides [8,10], anthocyanins [10], and crocins. Therefore, it is more reasonable and feasible to use all the waste saffron floral residues, including the petals and stamens, rather than just the petals, from the perspective of resource reuse. Menghini et al. demonstrated that extracts from tepals and stamens have protective effects on inflammation and oxidative stress [5]. Additionally, the extract from saffron floral bio-residues exhibited anti-oxidative and anti-hyperuricemic activities [16,17].

A previous study reported that the ethanolic extract of *C. sativus* petals reduced fasting blood glucose levels in STZ-induced diabetic rats [15]. This result suggested that the floral bio-residues may have a hypoglycemic effect. However, it is still unclear whether stigmaless floral residues of *C. sativus* (FRC) have the same effect. Precise regulation of circulating glucose is vital for maintaining human health. Skeletal muscle plays a pivotal role in maintaining glucose homeostasis through glucose uptake via insulin-dependent and -independent pathways [18]. Under normal physiological conditions, skeletal muscle serves as the major tissue for glucose transport, which is the rate-limiting step in glucose utilization. Furthermore, it is the primary site for glucose uptake in the postprandial state in humans [18]. Thus, we chose to use C2C12 myotubes to evaluate the effect of FRC on glucose metabolism in a preliminary study. In this study, we challenge the conventional focus on petals and argue for the comprehensive utilization of all saffron floral residues, including petals and stamens. Our study is novel in its approach to resource reuse and its focus on the glucose utilization promotion of stigmaless floral residues of *C. sativus* (FRC). We optimized the conditions for extraction of FRC flavonoids and further purified them with macroporous resin. We performed extraction optimization using a single-factor experiment, Plackett–Burman design, steepest ascent design, and response surface methodology (SRM). Subsequently, the crude extract obtained from ethanol extraction was purified using macroporous resin. The resin screening and adsorption kinetics were first investigated, and then the purification parameters were optimized through static and dynamic adsorption and desorption experiments. Then, a UPLC-MS/MS assay was performed to measure the main compounds of flavonoids and phenols in flavonoid-enriched extract from stigmaless floral residues of *C. sativus* (FFRC). Finally, we evaluated the effect of FFRC on glucose consumption and glucose uptake in C2C12 myotubes.

## 2. Results and Discussion

### 2.1. Single-Factor Experimental Results

The effects of the ethanol concentration, temperature, extraction time, solid-to-liquid ratio, and extraction times on the extraction yield of total flavonoids from the FRC were investigated with single-factor experiments.

#### 2.1.1. Ethanol Concentration

**Figure 1:**
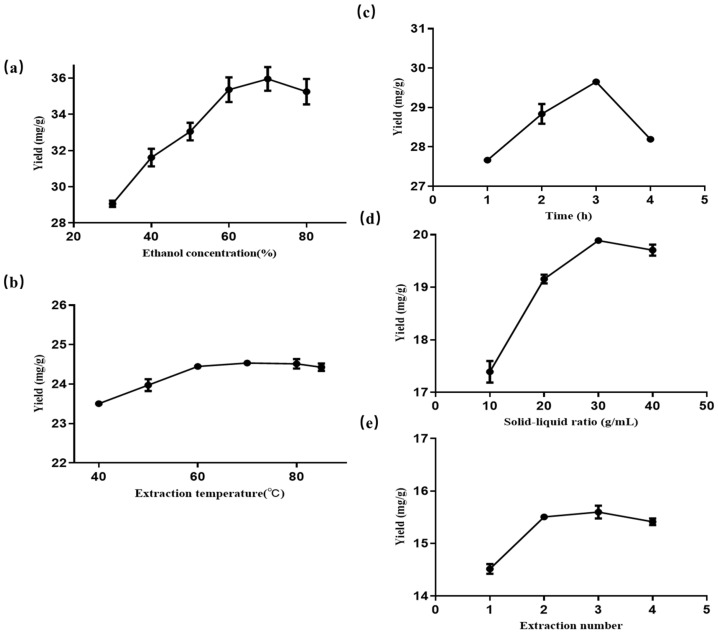
The effect of each single factor on the extraction efficiency of the total flavonoids from the FRC. (**a**) Ethanol concentration. (**b**) Temperature. (**c**) Time. (**d**) Solid-liquid ratio. (**e**) Extraction number. Results are expressed as the mean ± SD of three independent experiments, *n* = 3.

According to the results in Figure 1a, the extraction yield of flavonoids gradually increased as the ethanol concentration increased from 30% to 70%, with the highest yield at 70% ethanol concentration. However, when the ethanol concentration exceeded 70%, the extraction yield of total flavonoids began to decline with the increase in concentration. This can be explained by the fact that a higher ethanol concentration improves the permeability of solvent to the material but also increases the solubility of some non-polar substances that interfere with the extraction process [19]. Therefore, based on these results, 70% ethanol was chosen as the optimal extraction solvent in this single-factor experiment.

#### 2.1.2. Extraction Temperature

As shown in Figure 1b, the extraction yield of total flavonoids increased significantly when the extraction temperature rose from 40 to 70 °C. However, the yield decreased when the temperature exceeded 70 °C. This might be because higher temperatures enhanced the molecular movement, penetration, dissolution, and diffusion, which facilitated the release of flavonoids [20] but also caused the oxidation and degradation of flavonoids due to their thermal instability [21]. Therefore, 70 °C was chosen as the optimal extraction temperature.

#### 2.1.3. The Time of Extraction

The effect of extraction time (1 to 4 h) on the extraction yield of total flavonoids is shown in Figure 1c. The yield increased as the extraction time extended from 1 to 3 h. However, the yield decreased when the extraction time was longer than 3 h. This might be because prolonged heating at a high temperature would degrade some flavonoids [22]. Therefore, 3 h was determined as the optimal extraction time.

#### 2.1.4. Solid-to-Liquid Ratio

As the results show in Figure 1d, the extraction yield of total flavonoids increased with the solid-to-liquid ratio and reached a maximum value at 1:30. However, the yield decreased when the solid-to-liquid ratio was higher than 1:30. This might be because adding more solvent increased the amount of dissolved impurities and reduced the concentration of total flavonoids in the solution [23]. Therefore, 1:30 was selected as the optimal solid-to-liquid ratio.

#### 2.1.5. Extraction Times

Figure 1e shows that the extraction yield of total flavonoids increased significantly when the number of extraction times increased from 1 to 2, but only slightly when the number of extraction times increased to 3. Therefore, to save energy and time, the optimal number of extraction times was chosen to be 2.

### 2.2. Optimization by Plackett–Burman Experiment

Plackett–Burman designs are a type of fractional factorial designs that are used to screen a large number of factors to identify the ones that have a significant effect on the response [24]. The Plackett–Burman experiment can be used to quickly distinguish the factors with greater influence from a large number of potential factors with a minimum number of experiments, which can provide a reference for the final response surface experiments. We used the Plackett–Burman design to screen the significant factors affecting the extraction of total flavonoids from the FRC. Table 1 shows the experimental design and the corresponding response values. Table 2 shows the results of the regression analysis. As the results show in Table 2, the *p*-value of the regression model was 0.0069, which was less than 0.01, indicating that the model was highly significant. The effects of each factor on the extraction yield were ranked as follows: ethanol concentration (X_1_) > temperature (X_2_) > solid-to-liquid ratio (X_4_) > extraction time (X_3_). The ethanol concentration had a very significant effect on the response values (*p*-value: 0.0018 < 0.01), while the temperature had a significant effect (*p*-value: 0.0343 < 0.05). The other two factors, extraction time and solid-to-liquid ratio, were not significant (*p*-values: 0.2676 and 0.0879, respectively). Therefore, ethanol concentration and temperature were selected as the significant factors for the next optimization experiments using the steepest ascent method and response surface methodology (RSM). Based on the results and regression analysis (Table 2), a regression equation was obtained to represent the relationship between the yield of flavonoids and the four independent variables:

Y = 29.75 + 1.14X_1_ + 0.62X_2_ + 0.24X_3_ + 0.47X_4_

where Y represents the response value (extraction yield of total flavonoids). The model had a coefficient of determination (R^2^) of 0.8368, which indicated that the model could explain most of the data variability. The coefficient estimates of ethanol concentration and temperature were positive (1.14 and 0.62, respectively), implying that increasing these factors would increase the extraction yield of the total flavonoids.

### 2.3. Optimization by Steepest Ascent Experiment

The steepest ascent method is a type of gradient descent algorithm that finds the local minimum of a differentiable function by taking repeated steps in the opposite direction of the gradient [25]. It is suitable for determining the optimal ranges of the factors. Based on the Plackett–Burman analysis, ethanol concentration (X_1_) and extraction temperature (X_2_) were the most significant factors affecting the extraction yield of flavonoids. We applied the steepest ascent method to determine the approximate optimal ranges of the key factors for the extraction of total flavonoids from the FRC. As shown in Table 3, with the increase in ethanol concentration and extraction temperature, the flavonoid yield shows a trend of first increasing and then decreasing. The highest yield was at an ethanol concentration of 70% and a temperature of 70 °C (Run 4). However, with the further increase of ethanol concentration and extraction temperature, the yield decreased at an ethanol concentration of 80% and a temperature of 80 °C (Run 5). This can be explained by the fact that an increase in ethanol concentration improves the permeability of the material, but when the ethanol concentration exceeds a certain range, it can lead to an increase in some liposoluble components and other impurities, which is not conducive to the extraction of flavonoids [20]. Also, as the extraction temperature gradually increases, the flavonoid content in the solution also gradually increases, but after 70 °C, the flavonoid content begins to decrease. This may be because the temperature is too high, causing a small amount of flavonoid substances to become unstable, leading to a slight decrease in yield [21,22]. Similar results have been obtained in other studies [20,21,22].

Therefore, the optimal point obtained in this experiment, where the ethanol concentration was 70% and the extraction temperature was 70 °C, was used as the center point of the central composite design (CCD).

### 2.4. Optimization by Response Surface Methodology

In this study, we applied the response surface methodology (RSM) to optimize the ethanol extraction of flavonoids from FRC. Based on the principle of RSM, the best value derived for the factors from the path of the steepest ascent determination is used as the center point of the response surface, and a response analysis of the key factors is then carried out [26,27]. The CCD was used to study the interactions among the significant factors and to optimize their levels for the extraction of total flavonoids from the FRC. As the data shows in Table 4, the highest yield of flavonoids (24.40 mg/g) was obtained in run 4. A second-order polynomial model was fitted to describe the relationship between the extraction yield and the independent factors, ethanol concentration (X_1_), and extraction temperature (X_2_). The model equation is as follows:

Yield of flavonoids = 23.52 − 0.47 × X_1_ − 0.46 × X_2_ − 0.37 × X_1_X_2_ − 0.82 × X_1_^2^ − 0.77 × X_2_^2^

where Y is the predicted response, X_1_ and X_2_ are the input variables, 23.52 is a constant, −0.47 and −0.46 are linear coefficients, −0.37 is cross product coefficient, and −0.82 and −0.77 are quadratic coefficients.

The ANOVA results for the model are presented in Table 5. The model had a low *p*-value of 0.0108, which was less than 0.05, indicating that the model was statistically significant. The coefficient of determination (R^2^) was 0.8384, which indicated a good fit of the model to the experimental data. Therefore, this model could be used to predict the extraction yield of total flavonoids from the FRC under different conditions.

**Figure 2:**
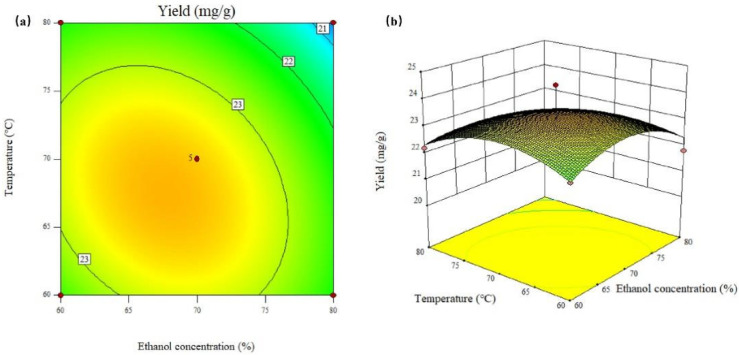
The response surface contour diagram (**a**) and the 3D surface map (**b**) for the interaction of ethanol concentration and temperature on the extraction efficiency of the total flavonoids from the FRC.

The 2D contour plot and 3D response surface curve are graphical representations of the regression equation (Figure 2). They show the effects of the independent factors, ethanol concentration (X_1_), and extraction temperature (X_2_) on the extraction yield of total flavonoids from the FRC. When the contours are elliptical or closely spaced, it indicates that there is a significant interaction between the two factors [28]. When the response surface is steep, it indicates that the response value is sensitive to the changes in the factor levels [29]. Figure 2 shows the contour plot (a) and the surface plot (b) for the extraction yield of flavonoids. The contours are elliptical, which means that the interaction between ethanol concentration and temperature is important. Figure 2a,b also shows that the maximum yield of 23.626 mg/g could be achieved at 67.7% ethanol concentration and 67.6 °C extraction temperature.

### 2.5. Validation of the Experiment

The quadratic model predicted that the optimal values of ethanol concentration and temperature were 67.7% and 67.6 °C, respectively. To verify the model, experiments were conducted at these optimal levels. The results showed that the average extraction yield of flavonoids from the FRC was 23.606 ± 0.082 mg/g (23.61943 mg/g, 23.51754 mg/g, and 23.68056 mg/g), which was close to the predicted value of 23.626 mg/g. The good agreement between the predicted and experimental results validated the reliability and accuracy of the RSM model. Therefore, the optimal conditions for extracting flavonoids from the FRC were established, and a crude extract rich in flavonoids was prepared accordingly.

### 2.6. Screening of Macroporous Resins for the Purification of Flavonoids from FRC

Macroporous resins adsorb based on the physical process of van der Waals forces and hydrogen bonds between the adsorbate and the adsorbent. Additionally, the adsorption and desorption efficiency of these resins is significantly influenced by their chemical structures, polarities, surface areas, and pore diameters [30,31]. In this study, we aimed to enrich the flavonoids from the crude ethanol extract of FRC. We screened six types of macroporous resins under the same experimental conditions and measured their static adsorption and desorption capacities. Table 6 shows the results of this screening. We found that HPD100 resin had the best adsorption and desorption effects on the flavonoids of FRC compared to other resins. Therefore, we selected HPD100 resin as the optimal resin for further optimization.

### 2.7. Static Adsorption and Desorption Kinetics on Resin HPD100

**Figure 3:**
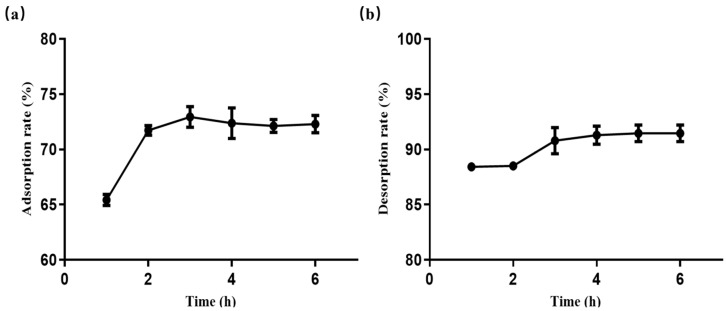
Kinetic curves for static adsorption (**a**) and desorption (**b**) of flavonoids from FRC on HPD100 macroporous resin. Results are expressed as the mean ± SD of three independent experiments, *n* = 3.

To further investigate the adsorption and desorption characteristics of flavonoids in the crude extract by HPD100 macroporous resin, experiments of static adsorption and desorption kinetics were performed. As the results show in Figure 3a, the adsorption rate of HPD100 experienced a rapid increase in the initial 2 h, followed by a slower increase until it reached equilibrium at the 3 h mark. Beyond this point, no further increase in adsorption rate was observed, establishing 3 h as the optimal adsorption time. The swift initial adsorption rate can be attributed to the adsorption taking place in the readily accessible mesopores of the particles. The subsequent slower uptake indicated a process with substantial mass transfer resistance within the particle [32]. Figure 3b shows the static desorption kinetic curve of flavonoids from FRC with HPD100 resin. The desorption rate underwent significant changes in the first 4 h. Past the 4 h mark, the desorption rate experienced a slow change and eventually stabilized, reaching equilibrium at 4 h. Hence, the optimal desorption time was determined to be 4 h.

### 2.8. Dynamic Adsorption and Desorption

#### 2.8.1. Effect of Concentration of Sample Solution on the Adsorption Capacity

**Figure 4:**
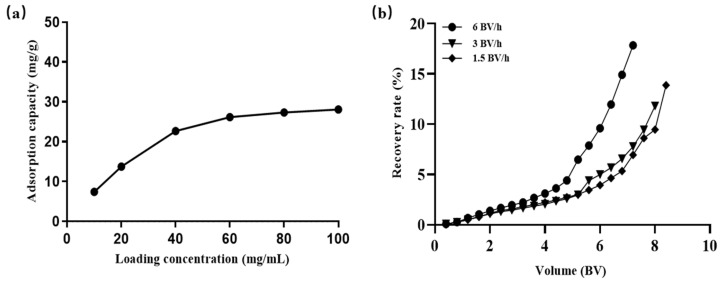
Factors affecting the adsorption properties of HPD100 resin. (**a**) Effect of sample concentration on adsorption capacity. (**b**) Effect of flow rate on adsorption capacity. Results are expressed as the mean ± SD of three independent experiments, *n* = 3.

As illustrated in Figure 4a, the adsorption capacity of flavonoids accelerated with an increase in the loading sample concentration. A rapid surge in adsorption capacity is observed when the loading concentration ranges from 10 to 40 mg/mL. However, upon reaching a loading sample concentration of 60 mg/mL, the growth in adsorption capacity decelerated and eventually stabilized. This can be attributed to the fact that as the concentration augments, the resin, not yet saturated, has ample space to adsorb flavonoids from the solution, thereby enhancing the adsorption capacity. Conversely, when the sample concentration is excessively high, the increase in impurities diminishes the available active sites on the resin. This leads to competition between the target substance and impurities for the active sites, resulting in a reduced adsorption capacity. Concurrently, the adsorption capacities at 40, 60, and 80 mg/mL are, respectively, 22.65 ± 0.38, 26.16 ± 0.14, and 27.19 ± 1.34 mg/g. The adsorption rate stands at 71.12% ± 0.88 at a loading concentration of 40 mg/mL and 55.78% ± 0.22 at 60 mg/mL. In order to strike a balance between the adsorption capacity and the adsorption rate, 40 mg/mL is selected as the optimal/ideal loading sample concentration.

#### 2.8.2. Effect of Flow Rate on the Adsorption Capacity

Figure 4b demonstrates the impact of the sample flow rate on the adsorption of flavonoids by the HPD100 resin. As the loading flow rate decreases, there is an increase in the quantity of flavonoids adsorbed by the macroporous resin. This, however, also results in a substantial increase in the loading adsorption time. This phenomenon can be attributed to the fact that a higher sample flow rate reduces the contact time between the target compound and the resin, causing some of the compound to exit the column before being adsorbed. This results in overload leakage and a decrease in the resin’s adsorption efficiency [33,34]. Consequently, a lower flow rate is favorable for the resin’s full adsorption, but it also extends the adsorption time and the experimental cycle in practice. To strike a balance between the adsorption capacity and the loading time, the loading flow rate was set at 3 BV/h. The leakage point is considered to have been reached when the flavonoid content in the effluent reaches 10% of the feed liquid concentration [35]. Under these conditions, the corresponding loading volume was determined to be 7 BV.

#### 2.8.3. Effect of Ethanol Concentration on Desorption Capacity

**Figure 5:**
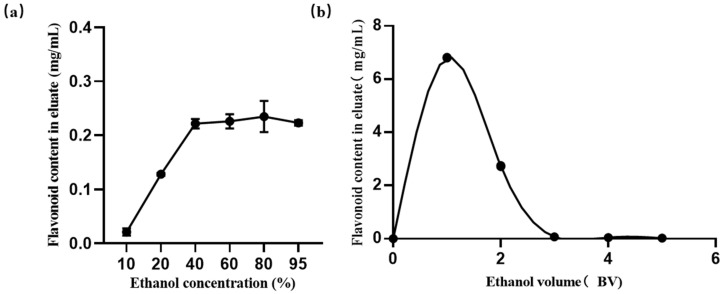
Factors affecting the desorption properties of HPD100 resin. (**a**) Effect of ethanol concentration on desorption capacity. (**b**) Effect of elution volume on desorption capacity. Results are expressed as the mean ± SD of three independent experiments, *n* = 3.

To identify the most effective ethanol content for the desorption process in HPD100 resin, we carried out experiments with a range of ethanol concentrations. As depicted in Figure 5a, the desorption capacity reached its zenith with 80% (*v*/*v*) ethanol, which proved to be highly efficient in eluting the flavonoids from the resin. Interestingly, an increase in ethanol concentration to 95% led to a decline in desorption capacity. As a result, 80% ethanol emerged as the optimal concentration for elution, owing to its superior desorption performance.

#### 2.8.4. Effect of Elution Volume on Desorption Capacity

Our investigation centered on the influence of the elution volume on the desorption capacity of flavonoids from the HPD100 resin. As illustrated in Figure 5b, the concentration of flavonoids in the eluate peaked at an elution volume of 1 BV, and no flavonoids were detected in the eluate when the elution volume reached or exceeded 4 BV. Consequently, 4 BV was selected as the optimal elution volume.

### 2.9. Verification of Optimum Conditions

Through static and dynamic experiments, the optimum purification conditions were obtained. HPD100 macroporous resin was chosen and loaded with a 40 mg/mL solution of crude extract. The loading volume was set at 7 BV, with a flow rate of 3 BV per hour. Following this, the resin, which had adsorbed the crude extract, was rinsed with distilled water until the Molisch reaction turned negative. Subsequently, the column resin was eluted with 80% ethanol at a flow rate of 3 BV/h. The 80% ethanol eluent was then concentrated and freeze-dried to yield the final product, FFRC.

Validation experiments were conducted in line with the process conditions identified above. The results demonstrated a significant increase in the content of flavonoids, from 23.606 ± 0.082 mg/g in the crude ethanol extract to 128.297 ± 4.642 mg/g in the FFRC. This represents an approximate 5.435-fold increase post-purification. These findings suggest that the method is highly efficient and effective in separating and purifying flavonoids from the crude ethanol extract of FRC. Thus, it is suitable for the purification of flavonoids in the crude ethanol extract of FRC.

The solvent extraction method combined with macroporous resin purification used in this study offers several advantages over other methods, such as supercritical fluid extraction and ultrasound-assisted extraction [22,36]. Firstly, solvent extraction is a simple, cost-effective, and efficient method for extracting flavonoids from natural sources [37]. It allows for the extraction of a wide range of flavonoids, including those that might not be efficiently extracted by other methods [36]. Secondly, the use of macroporous resin for purification allows for the selective adsorption and desorption of flavonoids, resulting in high purity extract [36]. This method is also scalable, making it suitable for large-scale production [36]. In comparison, other extraction methods such as supercritical fluid extraction and ultrasound-assisted extraction, while effective, can be more complex and costly [22,36]. They may also require specialized equipment and may not be suitable for all types of flavonoids or for large-scale production [22,36]. Similarly, other purification methods such as column chromatography and membrane separation, while offering high selectivity, can be time-consuming, costly, and may not be easily scalable [38].

### 2.10. Analyses of Flavonoids and Phenolic Compounds by LC-MS/MS

**Figure 6:**
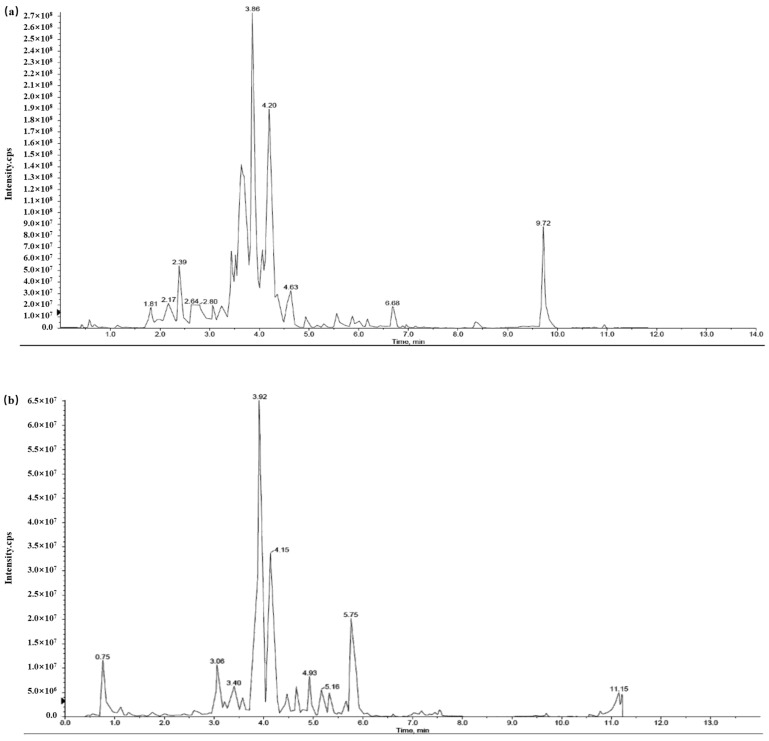
Total ion flow chromatogram of FFRC. (**a**) Total ion flow chromatogram in positive ion mode. (**b**) Total ion flow chromatogram in negative ion mode.

The flavonoids and phenolic compounds in FFRC were analyzed using LC-MS/MS by Wuhan Metware Biotechnology Co., Ltd., Wuhan, China. The analysis was conducted in both ESI-positive and ESI-negative ion modes. The Metware Database (MWDX) was utilized to identify the compounds, leading to the detection of a total of 529 flavonoids and phenolic compounds. The Total ion flow chromatograms in both positive and negative ion modes are illustrated in Figure 6.

Out of the identified compounds, 99 exhibited a relative peak area greater than 0.1%. These compounds are detailed in Supplementary Table S1. The results revealed that the primary flavonoids in FFRC were classified as kaempferol glycosides, isorhamnetin glycosides, and quercetin glycosides.

Specifically, 19 variants of kaempferol glycosides were identified, contributing to a total relative peak area of 18.48%. The most abundant among these were Kaempferol-3- *O* -glucoside (Astragalin) at 3.10%, kaempferol 3- *O* -(2- *O* -acetylglucosyl) glucoside at 2.81%, and kaempferol 3- *O* -(6- *O* -acety1) glucopyranoside-7- *O* -glucopyranoside at 2.72%.

Seven variants of isorhamnetin glycosides were identified, accounting for a total relative peak area of 12.63%. The most abundant were Isorhamnetin-7- *O* -glucoside (Brassicin) at 2.71% and Isorhamnetin-3- *O* -Glucoside at 2.49%.

Eight variants of quercetin glycosides were identified, contributing to a total relative peak area of 9.47%. The most abundant were Quercetin-3- *O* -sophoroside (Baimaside) at 2.71%, Quercetin-3- *O* -glucoside (Isoquercitrin) at 1.46%, and Quercetin-3- *O* -galactoside (Hyperin) at 1.44%.

### 2.11. FFRC Promoted Glucose Consumption in C2C12 Myotubes

**Figure 7:**
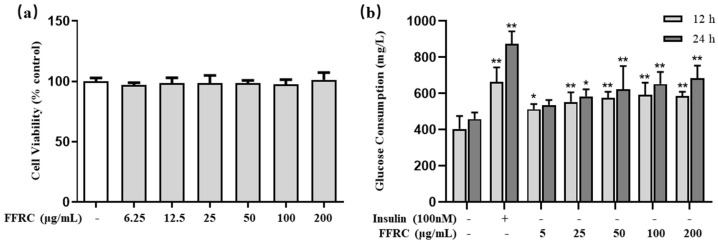
FFRC enhanced glucose consumption in C2C12 myotubes. (**a**) Cell viability was assessed using an MTT assay after co-treatment of myotubes with FFRC for 24 h. (**b**) Glucose consumption was quantified by glucose oxidase assay after co-treatment myotubes with FFRC for 12 or 24 h. Results are expressed as mean ± SD (*n* = 6). * *p* < 0.05, ** *p* < 0.01, vs. control group.

The cytotoxic effect of FFRC on C2C12 myotubes was assessed using an MTT assay. As the results show in Figure 7a, the survival rate of the myotubes was found to be over 90% after a 24 h treatment with FFRC. This finding suggests that FFRC does not impair cell viability and exhibits no cytotoxicity at the tested concentrations, thereby confirming its suitability for further investigations. As a result, FFRC concentrations of less than 200 μg/mL were selected for the subsequent glucose consumption assay. As the results show in Figure 7b, FFRC significantly enhanced the glucose consumption of myotubes in a dose-dependent manner, regardless of the duration (12 h or 24 h) of FFRC treatment (*p* < 0.05 or 0.01).

### 2.12. FFRC Enhanced Glucose Uptake in C2C12 Myotubes

**Figure 8:**
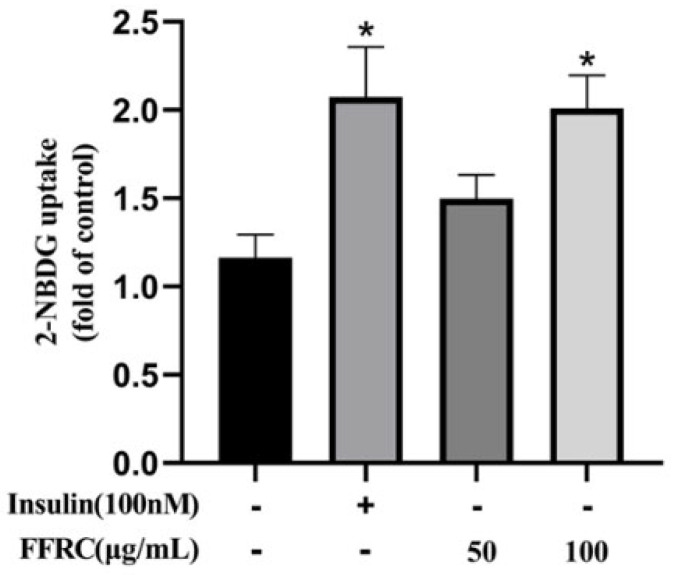
FFRC stimulated glucose uptake in C2C12 myotubes. After incubation of myotubes with FFRC (50 and 100 μg/mL) for 3 h or insulin (100 nmol/L) for 30 min, glucose uptake was measured using the 2−NBDG method. Values are expressed as mean ± SD (*n* = 3). * *p* < 0.05 vs. control group.

Then, a 2-NBDG uptake assay was employed to determine the effect of FFRC on glucose uptake. The results show that FFRC at concentrations of 50 and 100 μg/mL significantly enhanced glucose uptake in C2C12 myotubes by 28.75 and 72.62%, respectively, compared to the controls (*p* < 0.01, Figure 8).

These findings indicated that FFRC significantly stimulated glucose uptake in C2C12 myotubes. The underlying mechanism is speculated to involve the activation of the PI3K/AKT and AMPK pathways, both of which play pivotal roles in regulating glucose uptake in skeletal muscle [39,40]. The PI3K/AKT pathway is typically stimulated by insulin via the insulin receptor substrate 1 (IRS1), which leads to the translocation of GLUT4 to the cell membrane and subsequent glucose uptake. Conversely, the AMPK pathway is activated by muscle contractions and exercise, thereby promoting glucose uptake independently of insulin [41]. Thus, the observed effects of FFRC could potentially be attributed to its role in modulating these key signaling pathways, thereby enhancing glucose uptake and consumption. Nevertheless, further in vitro and in vivo studies are required to validate these findings.

The demonstrated ability of FFRC to enhance glucose uptake and consumption suggests its potential as a functional food ingredient for managing blood glucose levels, thereby indicating its possible utility as a natural hypoglycemic agent. Considering the rising prevalence of metabolic disorders such as type 2 diabetes, which is often linked with impaired glucose uptake in skeletal muscle, FFRC could provide a novel dietary approach for improving glucose homeostasis. Nevertheless, further research is required to investigate these potentials in vivo and to explore the applicability of FFRC in real-world scenarios.

## 3. Material and Methods

### 3.1. Materials and Chemicals

The stigmaless floral residues of *Crocus sativus* L., from Chongming, Shanghai, identified by Prof. Li Hongqing, School of Life Sciences, East China Normal University. Kaempferol (purity > 98%), obtained from Dalian Meilun Biotechnology Co., Ltd. (Dalian, China). Ninety-five percent ethanol was obtained from Shanghai Titan Technology Co., Ltd. (Shanghai, China). HPD100, HPD600, HPD826, D101, NKA-9, and ADS-17 were purchased from Cangzhou Bon Adsorber Technology Co., Ltd. (Cangzhou, China). FA-free bovine serum albumin (BSA) and insulin (bovine) were purchased from Yeasen Biotechnology (Shanghai, China). 3-(4,5-dimethylthiazol-2-yl)-2,5-diphenyltetrazolium bromide (MTT) and dimethyl sulfoxide (DMSO) were purchased from Sigma-Aldrich Chemical Co. (St. Louis, MO, USA). Fetal bovine serum (FBS) was purchased from Bovogen Biologicals (East Keilor, VIC, Australia). Other cell culture materials, including Dulbecco’s modified eagle’s medium (DMEM); horse serum; and antibiotic, antimycotic, and trypsin solutions were obtained from Gibco (Gaithersburg, MD, USA). Glucose oxidase determination kit was obtained from Shanghai Rongsheng Bio-Pharmaceutical Co., Ltd. (Shanghai, China).

### 3.2. Measurement of Total Flavonoids Content

The FRC were rich in flavonol glycosides, especially kaempferol glycosides [10,42]. The total flavonoid content was determined with a UV assay using kaempferol as a standard, following a previous report [43]. A kaempferol powder (1 mg) was weighed accurately and dissolved in methanol to prepare a 1 mL solution. Then, 0.2 mL of this solution was diluted with methanol to 5 mL, obtaining a 0.04 mg/mL kaempferol solution. Next, six aliquots of this solution (0.1 mL, 0.2 mL, 0.4 mL, 0.6 mL, 0.8 mL, and 1 mL) were taken and diluted to 1 mL with methanol, resulting in kaempferol standard solutions with concentrations of 0.004 mg/mL, 0.008 mg/mL, 0.016 mg/mL, 0.024 mg/mL, 0.032 mg/mL, and 0.04 mg/mL respectively. Finally, the absorbance of each standard solution was measured at 365 nm and a standard curve of absorbance versus concentration was plotted. The flavonoid content was calculated from the regression equation: y = 54.726x + 0.0528 (R^2^ = 0.9999), where y is the absorbance and x is the content (mg/mL). The extraction yield of the total flavonoids was calculated from the equation given below:

$$\mathrm{Extraction}\;\mathrm{yield}\;\mathrm{of}\;\mathrm{flavonoids}\;(\mathrm{mg}/g)=\frac{C\times V}{m}\times 100\%$$

where C represents the flavonoid content (mg/mL), V represents the total volume of the extract (mL), and m represents the weight of the material (powder of FRC, g).

### 3.3. Single-Factor Experimental Design

The effects of ethanol concentration, extraction temperature, extraction time, solid-to-liquid ratio, and extraction frequency on the extraction yield of flavonoids were investigated using a single-factor experimental design. The experiments were performed according to the procedure outlined in Table 7.

### 3.4. Plackett–Burman Experimental Design

To screen the significant variables among the four factors of ethanol concentration, extraction temperature, extraction time, and solid-to-liquid ratio, a Plackett–Burman experimental design was applied based on the results of the single-factor experiment. The yield of flavonoids was set as the response variable, and each factor was assigned two levels of +1 (high level) and −1 (low level). Table 1 shows the levels and codes of the factors for the Plackett–Burman design. A total of 12 experiments were conducted. The Design-Expert 10.0.7 software was used for the design and analysis of the Plackett–Burman experiment.

### 3.5. The Experiment of the Steepest Ascent Path

To find the optimal parameters for the extraction yield of total flavonoids, experiments for each response were performed along the path of the steepest ascent experiment with defined intervals. The significant factors (ethanol concentration and extraction temperature) were adjusted stepwise by increasing or decreasing them based on the coefficients of the Plackett–Burman experiment results. The ethanol concentration and extraction temperature were set at 40% and 40 °C, respectively, as the starting points, and changed by 10% and 10 °C for each step. The other non-significant factors were fixed at intermediate values (extraction time of 3 h and solid-to-liquid ratio of 1:30). The point that yielded the maximum extraction was close to the optimal parameters and was chosen as the center point in the central composite design (CCD).

### 3.6. Central Composite Design (CCD) Experiment

We conducted a central composite design (CCD) in the optimum vicinity to locate the true optimum of ethanol concentration (X_1_) and extraction temperature (X_2_) for the extraction efficiency of flavonoids. The CCD was generated with Design-Expert 10.0.7 software. According to this design, 13 experiments were performed, with five replicates at the center point for estimating the experimental error variance. In this study, two key variables with five levels were adopted (given in Table 4). We measured the extraction efficiency of flavonoids for each trial and fitted the results to a second-order polynomial model. Then, we evaluated the statistical significance of the model equation using Fisher’s test (F value) and the proportion of variance explained by the model by the multiple coefficients of determination (R^2^ value).

### 3.7. Validation of the Experiment Design

The optimal levels of the significant factors were determined with the predicted optimal value from the RSM experiment results. The other factors were kept at the optimal levels obtained from the single-factor experiment. The extraction efficiency of flavonoids under these conditions was then measured.

### 3.8. Purification of Flavonoids with Macroporous Resins

#### 3.8.1. Pretreatment of Macroporous Resins

The resins were soaked in a solution of 95% (*v*/*v*) ethanol, equivalent to twice the volume of the resins, for 24 h. The ethanol was then filtered out and the resins were rinsed with deionized water to remove any traces of alcohol. Then, the washed resins were immersed with 5% HCl and 5% NaOH, respectively, for 4 h. Lastly, they were washed with deionized water until the pH of the filtrate reached neutrality and were soaked in deionized water at room temperature for standby.

#### 3.8.2. Static Adsorption and Desorption Experiments on Macroporous Resins for the Purification of Total Flavonoids

Six types of macroporous resins (HPD100, HPD600, HPD826, D101, NKA-9, and ADS-17) were each weighed to be 1 g and then added separately into six 100 mL Erlenmeyer flasks. The resins in each flask were soaked in 20 mL of crude flavonoid-rich extract solutions with an initial content of 40 mg/mL. Then, the flasks were shaken using a shaking incubator with a speed of 120 rpm at 25 °C for 24 h. After absorption equilibrium was reached, the resins were filtered and the concentration of total flavonoids in the filtrate was determined. Subsequently, the remaining resin was washed with deionized water until the eluents were colorless. Then, 30 mL of 60% ethanol was added to the flasks for desorption. The flasks were shaken with a speed of 120 rpm at 25 °C for 24 h again. After desorption equilibrium was reached, the solutions were filtered and the total flavonoid contents of the supernatant were measured. The corresponding adsorption and desorption capacity and ratio of each resin were calculated using the equations below [44]:

$$Q_{1}=\frac{\left(C_{0}-C_{e}\right)\times V_{0}}{M}$$

$$E_{1}(\%)=\frac{C_{0}-C_{e}}{C_{0}}\times 100\%$$

$$Q_{2}=\frac{C_{d}\times V_{2}}{M}$$

where Q_1_ and Q_2_ are the adsorption and desorption capacity (amount) at equilibrium (mg/g), respectively; C_0_ and C_e_ are the original and adsorption equilibrium concentrations of the total flavonoids in the solutions (mg/mL), respectively; V_0_ is the initial sample volume added into the flask (mL); M is the dry weight of the resin (g); C_d_ is the equilibrium concentration of total flavonoids in the desorption solution (mg/mL); V_2_ is the desorption solution volume (mL); and E_1_ (%) represents the adsorption rate.

#### 3.8.3. Adsorption and Desorption Kinetics on Selected Resin

Static adsorption kinetics experiment: According to adsorption and desorption capacities, we selected the HPD100 resin for the adsorption kinetics study. We added 1 g of pretreated resin HPD100 in a 100 mL Erlenmeyer flask to 30 mL of crude flavonoid-rich extract solution (40 mg/mL) and then shook it at 25 °C and 120 rpm. We took out 500 μL of supernatants at specific time intervals (1 h) until equilibration and determined the concentrations of total flavonoids in the supernatants to plot the static adsorption kinetics curve.

Static desorption kinetic experiment: After completing the adsorption experiment, we collected the HPD100 resins and dried the surface water with filter paper. Subsequently, we immersed 0.5 g of resin saturated with adsorption in 30 mL of 70% ethanol (*v*/*v*) and incubated it at 25 °C and 120 rpm. We took out 500 μL of supernatants at specific time intervals (1 h) until equilibration and measured the concentrations of total flavonoids in the supernatants to plot the static desorption kinetics curve.

#### 3.8.4. Effect of Sample Concentrations on Adsorption Efficiency

First, we added six aliquots (accurately weighed 1 g) of HPD100 macroporous resin separately into six 100 mL Erlenmeyer flasks. Next, we added 20 mL of sample solutions containing different concentrations of crude flavonoid-rich extracts (10, 20, 40, 60, 80, and 100 mg/mL). The flasks were continually shaken for 3 h at 120 rpm and 25 °C. We determined the original and adsorption equilibrium concentrations of total flavonoids in the solutions and then drew the adsorption concentration curve.

#### 3.8.5. Effect of Flow Rates on Dynamic Adsorption

The dynamic experiments of adsorption and desorption were performed in a chromatographic column (1:10), wet-packed with a column volume that was around 25 mL of the selected HPD100 resin. The solution of crude flavonoid-rich extract from FRC was filtered through a 0.45 µm membrane and then applied to the resin at a suitable flow rate. The solutions of crude extracts with a concentration of 40 mg/mL and a volume of 250 mL were loaded at different flow rates of 1.5, 3, and 6 BV/h (bed volume per hour). The effluent liquid was collected every 25 mL and the total flavonoid concentration was detected. Then, the flow rate–volume curves were plotted.

#### 3.8.6. Effect of Eluent Concentration on Desorption Efficiency

To determine the effect of eluent concentration on desorption efficiency, ethanol/water mixtures with different ratios (10, 20, 40, 60, 80, and 95% *v*/*v*) were used as solvents. An amount of 0.5 g of HPD 100 resins loaded with crude flavonoid extract were placed in conical flasks, and 20 mL of each ethanol solution was added. The flasks were shaken at 25 °C and 120 rpm for 4 h to reach equilibrium. Then, the solutions were filtered and the total flavonoids contents of the filtrates were measured. The elution concentration curve was plotted based on the results.

#### 3.8.7. Effect of Elution Volume on Dynamic Desorption

A glass column was packed with 25 mL of HPD100 macroporous resin, which had a diameter-to-height ratio of 1:10. The crude flavonoid-rich extract was loaded onto the resin column until the adsorption equilibrium was reached. Then, the deionized water was first used for washing the resin column and then eluted with 80% ethanol solution at a flow rate of 3BV/h. The eluent was collected in 25 mL fractions, and the total flavonoid concentration in each fraction was measured with UV spectrophotometry. The dynamic elution curve was drawn based on the elution volume and flavonoid content.

### 3.9. UPLC-ESI-MS/MS Analysis

Flavonoid and phenolic compounds in the extract were identified using UPLC-ESI-MS/MS. The extracts were sent to Mai Wei Biotechnology Co., Ltd. (Shanghai, China), and all UPLC-ESI-MS/MS analyses and data processing were performed by Mai Wei Biotechnology Co., Ltd.

### 3.10. Cell Culture and Differentiation

C2C12 mouse myoblasts were obtained from the National Center for Drug Screening (Shanghai, China). They were cultured according to the method described by Fu [45]. Briefly, myoblasts were cultured in DMEM containing 10% fetal bovine serum, 100 U/mL penicillin, and 100 U/mL streptomycin and maintained in an incubator at 37 °C and 5% CO_2_. Cells were then seeded on 96-well plates at a density of 5 × 10^4^ cells/mL. After about 24 h, when they reached around 70–80% confluence, the medium was changed to DMEM with 2% horse serum and was refreshed every 2 days. After 6–7 days, the differentiation of C2C12 mouse myoblasts into myotubes was completed and the experiments were started.

### 3.11. MTT Assay

An MTT assay was performed to evaluate the effect of FFRC on cell viability. C2C12 myoblasts were cultured in 96-well plates and their differentiation into myotubes was induced. After differentiation, the myotubes were then incubated in DMEM with 0.2% BSA for 6 h and treated with various concentrations of FFRC for 24 h. After that, 20 μL of 3 mg/mL MTT was added to each well and they were incubated for 2.5 h at 37 °C. The MTT formazan crystals were dissolved by adding 200 μL of dimethyl sulfoxide to each well and shaken until clear. Finally, the absorbance of each well was measured at 490 nm and cell viability was calculated using the following formula [45].

$$\mathrm{Cell}\;\mathrm{viability}\%=\frac{OD\mathrm{sample}-OD\mathrm{blank}}{OD\mathrm{control}-OD\mathrm{blank}}\times 100\%$$

### 3.12. Glucose Consumption Assay

Using the glucose oxidase method, glucose consumption of the myotubes was measured. C2C12 myoblasts were cultured in 96-well plates and differentiated into myotubes. They were then incubated in DMEM with 2% BSA for 6 h and exposed to phenol red-free DMEM with 0.2% BSA and various concentrations of FFRC for 12 and 24 h. The supernatant was removed and the glucose level of the medium was measured using a glucose oxidase assay kit following the manufacturer’s instructions. The difference between the initial and the final glucose levels was calculated as the glucose consumption of the cells.

### 3.13. 2-NBDG Uptake Assay

Cell glucose uptake was measured by the uptake of 2-NBDG, a fluorescent deoxyglucose analog. C2C12 myoblasts were differentiated into myotubes in black 96-well plates and treated with different FFRC concentrations for various durations. Before harvesting, the myotubes were rinsed with warm sterile PBS (37 °C) and incubated in glucose-free DMEM with 0.2% BSA for an hour. Then, the myotubes were washed with warm sterile PBS again and exposed to a medium with 80 μM 2-NBDG for 30 min. After another wash with warm sterile PBS, the fluorescence intensity of each well was measured at 485 nm excitation and 520 nm emission wavelengths. Cell glucose uptake was calculated using the following formula:

$$Glucose\;Uptake=\frac{FI_{Sample}-FI_{Blank}}{FI_{Control}-FI_{Blank}}$$

### 3.14. Statistical Analysis

All experiments were repeated at least three times independently, and the data were presented as the mean ± standard deviation (SD). The Design-Expert 10.0.7 software was utilized to analyze the experimental data related to the response surface design. Bioactivity analysis results were processed using IBM SPSS Statistics 23.0 software, with significant differences confirmed through one-way ANOVA and Duncan’s multiple range tests. A *p*-value less than 0.05 was deemed significant, while a *p*-value less than 0.01 was considered highly significant. All graphical representations were created using GraphPad Prism 8.0 software.

## 4. Conclusions

In this study, we systematically established a process route for the extraction and purification of total flavonoids from the FRC. The response surface methodology was used to optimize the extraction conditions, and the macroporous resin was applied for purification. The extraction conditions were optimized as follows: ethanol concentration of 67.7%, temperature of 67.6 °C, solid-to-liquid ratio of 1:30, extraction time of 3 h, and two extracts. Following extraction, the HPD100 macroporous resin was selected for purification. The optimum purification conditions were a loading volume of 7 BV, adsorption concentration of 40 mg/mL, adsorption flow rate of 3 BV/h, and 80% ethanol as the eluent with a volume of 4 BV. The culmination of this process was the production of FFRC with a flavonoid content of 128.30 ± 4.64 mg/g. We further explored the biological activity of FFRC and found that it significantly promoted glucose consumption and glucose uptake in C2C12 myotubes in vitro. In summary, this study not only provides a systematic and replicable method for the preparation of total flavonoids from the non-medicinal parts of saffron but also uncovers the activities of this extract. These findings pave the way for the industrial production and utilization of these resources, with potential implications for therapeutic applications and functional food development.

## Figures and Tables

**Table 1:** Plackett–Burman experiment design and response value.

Run	Levels	Variables				Yield (mg/g)
		Ethanol Concentration (%) X_1_	Temperature (°C) X_2_	Extraction Time (min) X_3_	Solid-to-Liquid Ratio (g/mL) X_4_	
	1	80	80	4	1:40	
	−1	40	40	2	1:20	
1		1	−1	−1	−1	29.53 ± 0.17
2		1	1	−1	−1	31.79 ± 0.13
3		1	−1	1	1	31.01 ± 0.23
4		1	1	1	−1	31.03 ± 0.20
5		1	−1	1	1	30.56 ± 0.23
6		1	1	−1	1	31.72 ± 0.06
7		−1	1	1	1	30.80 ± 0.53
8		−1	1	−1	1	28.93 ± 0.16
9		−1	−1	1	−1	28.86 ± 0.16
10		−1	1	1	−1	28.22 ± 0.23
11		−1	−1	−1	1	28.56 ± 0.32
12		−1	−1	−1	−1	26.56 ± 0.26

Note: X_1_: Ethanol Concentration; X_2_: Temperature; X_3_: Extraction Time; X_4_: Solid-to-Liquid Ratio. Results are expressed as the mean ± SD of three independent experiments, *n* = 3.

**Table 2:** Regression analysis of the Plackett–Burman experiment design.

Source	Coefficient Estimate	*F* Value	*p*-Value (Prob > *F*)	Ranking
Model	29.8	8.98	0.0069	Significant
X_1_	1.14	23.65	0.0018	1
X_2_	0.62	6.88	0.0343	2
X_3_	0.28	1.45	0.2676	4
X_4_	0.47	3.93	0.0876	3

Note: R^2^ = 0.8368; X_1_: Ethanol Concentration; X_2_: Temperature; X_3_: Extraction Time; X_4_: Solid-to-Liquid Ratio.

**Table 3:** The results of the path of steepest ascent.

Run	Ethanol Concentration (%) X_1_	Temperature (°C) X_2_	Yield (mg/g)
1	40	40	20.82 ± 0.52
2	50	50	22.00 ± 0.54
3	60	60	22.50 ± 0.90
4	70	70	23.04 ± 0.20
5	80	80	21.78 ± 0.09

Results are expressed as the mean ± SD of three independent experiments, *n* = 3.

**Table 4:** Design and results of CCD Central Composite response surface design.

Run	Ethanol Concentration (%) X_1_	Temperature (°C) X_2_	Yield (mg/g)
1	0 (70)	0 (70)	23.43 ± 0.11
2	0 (70)	0 (70)	23.32 ± 0.18
3	0 (70)	0 (70)	23.07 ± 0.19
4	0 (70)	0 (70)	24.40 ± 1.01
5	0 (70)	0 (70)	23.36 ± 0.37
6	0 (40)	−1.41 (55.86)	22.87 ± 0.28
7	1.41 (84.14)	0 (70)	21.86 ± 0.09
8	−1.41 (55.86)	0 (70)	22.52 ± 0.04
9	−1.41 (55.86)	−1.41 (55.86)	22.45 ± 0.19
10	1.41 (84.14)	−1.41 (55.86)	21.79 ± 0.04
11	−1.41 (55.86)	1.41 (84.14)	22.19 ± 0.16
12	1.41 (84.14)	1.41 (84.14)	20.04 ± 0.19
13	0 (70)	1.41 (84.14)	21.71 ± 0.23

Results are expressed as the mean ± SD of three independent experiments, *n* = 3.

**Table 5:** Analysis of variance for response surface quadratic model.

Sources Variation	Sum Squares	Degree of Square	Mean Square	*F* Value	*p*-Value (Probd > *F*)	Significant
Model	11.76	5	2.35	7.26	0.0108	*
X_1_	1.75	1	1.75	5.42	0.0528	
X_2_	1.68	1	1.68	5.18	0.0571	
X_1_X_2_	0.55	1	0.55	1.71	0.2324	
X_1_^2^	4.68	1	4.68	14.46	0.0067	**
X_2_^2^	4.11	1	4.11	12.69	0.0092	**
Residual	2.27	7	0.32			
Lack of Fit	1.23	3	0.41	1.57	0.3278	
Pure Error	1.04	4	0.26			
Cor Total	14.03	12				

Note: R^2^ = 0.8384; Cor Total: corrected total; significance: ** *p* < 0.01, * *p* < 0.05.

**Table 6:** Static adsorption and desorption results in different types of macroporous resins.

Type	Resin Properties	Adsorpti on Capacity (mg/g)	Desorption Capacity (mg/g)
HPD100	Non-polar	1.23 ± 0.03	0.69 ± 0.03
HPD600	Polar	0.64 ± 0.01	0.44 ± 0.01
HPD826	Hydrogen bond	0.85 ± 0.03	0.43 ± 0.01
D101	Non-polar	1.12 ± 0.05	0.63 ± 0.04
NKA-9	Polar	0.72 ± 0.03	0.22 ± 0.15
ADS-17	Middle polar	0.42 ± 0.01	0.09 ± 0.15

Results are expressed as the mean ± SD of three independent experiments, *n* = 3.

**Table 7:** Single-factor experimental design.

Single Factor	Ethanol Concentration (%)	Temperature (°C)	Extraction Time (min)	Solid-to-Liquid Ratio (g/mL)	Extraction Times
Single factor 1	30%	70 °C	1 h	1:20	1
	40%	70 °C	1 h	1:20	1
	50%	70 °C	1 h	1:20	1
	60%	70 °C	1 h	1:20	1
	70%	70 °C	1 h	1:20	1
	80%	70 °C	1 h	1:20	1
Single factor 2	70%	40 °C	1 h	1:20	1
	70%	50 °C	1 h	1:20	1
	70%	60 °C	1 h	1:20	1
	70%	70 °C	1 h	1:20	1
	70%	80 °C	1 h	1:20	1
	70%	85 °C	1 h	1:20	1
Single factor 3	70%	70 °C	1 h	1:20	1
	70%	70 °C	2 h	1:20	1
	70%	70 °C	3 h	1:20	1
	70%	70 °C	4 h	1:20	1
Single factor 4	70%	70 °C	1 h	1:10	1
	70%	70 °C	1 h	1:20	1
	70%	70 °C	1 h	1:30	1
	70%	70 °C	1 h	1:40	1
Single factor 5	70%	70 °C	1 h	1:20	1
	70%	70 °C	1 h	1:20	2
	70%	70 °C	1 h	1:20	3
	70%	70 °C	1 h	1:20	4

## Data Availability

The data are included in the figures and tables of this manuscript.

## Supplementary Materials

The following supporting information can be downloaded at: https://www.mdpi.com/article/10.3390/molecules29143271/s1, Table S1. LC-MS/MS analysis revealed the presence of flavonoids and phenolic compounds in FFRC.
