# Supplementary material for: Optimization of Extraction and Purification of Flavonoids from Stigmaless Floral Residues of Crocus sativus L. and Their Stimulatory Effect on Glucose Uptake In Vitro
Source: Molecules. 2024 Jul 10;29(14):3271. doi: 10.3390/molecules29143271 (PMC11279114; doi:10.3390/molecules29143271)
Supplement: Supplementary file 1 [file molecules-29-03271-s001.zip › molecules-3038885-supplementary.pdf]

Table S1. LC-MS/MS analysis revealed the presence of flavonoids and phenolic compounds in FFRC

Molecular Weight (Da)	Formula	Ionization model	Compounds	Area%
194.058	$C_{10}H_{10}O_4$	[M+H]+	Vanillin acetate	0.317
302.043	$C_{15}H_{10}O_7$	[M-H]-	Tricetin (5,7,3',4',5'-Pentahydroxyflavone)	0.127
308.126	$C_{16}H_{20}O_6$	[M+H]+	Toddalolactone	0.243
624.169	$C_{28}H_{32}O_{16}$	[M+H]+	Tamarixetin-3-*O*-glucoside-7-*O*-rhamnoside	2.101
316.058	$C_{16}H_{12}O_7$	[M+H]+	Tamarixetin (3,3',5,7-Tetrahydroxy-4'-Methoxyflavone)	0.144
452.131	$C_{21}H_{24}O_{11}$	[M-H]-	Sieboldin	0.153
624.169	$C_{28}H_{32}O_{16}$	[M+H]+	Sexangularetin-3-*O*-glucoside-7-*O*-rhamnoside	2.015
626.148	$C_{27}H_{30}O_{17}$	[M+H]+	Rhodioflavonoside	0.261
624.168	$C_{28}H_{32}O_{16}$	[M+H]+	Rhamnetin-3-*O*-Rutinoside	2.027
478.111	$C_{22}H_{22}O_{12}$	[M+H]+	Rhamnetin-3-*O*-Glucoside	2.620
464.095	$C_{21}H_{20}O_{12}$	[M-H]-	Quercetin-7-*O*-glucoside	1.119
464.095	$C_{21}H_{20}O_{12}$	[M-H]-	Quercetin-4'-*O*-glucoside (Spiraeoside)	1.193
626.148	$C_{27}H_{30}O_{17}$	[M+H]+	Quercetin-3-*O*-sophoroside (Baimaside)	2.708
464.095	$C_{21}H_{20}O_{12}$	[M-H]-	Quercetin-3-*O*-glucoside (Isoquercitrin)	1.464

464.095	$C_{21}H_{20}O_{12}$	[M+H]+	Quercetin-3-*O*-galactoside (Hyperin)	1.441
610.132	$C_{30}H_{26}O_{14}$	[M+H]+	Quercetin-3-*O*-(6"-*O*-p-Coumaroyl)galactoside	0.830
480.09	$C_{21}H_{20}O_{13}$	[M+H]+	Quercetagetin-7-*O*-glucoside	0.136
426.153	$C_{20}H_{26}O_{10}$	[M+H]+	Praeroside VI	0.160
148.016	$C_8H_4O_3$	[M+H]+	Phthalic anhydride	0.214
640.167	$C_{28}H_{32}O_{17}$	[M+H]+	Patuletin-3-*O*-rutinoside	0.356
610.153	$C_{27}H_{30}O_{16}$	[M+H]+	Orientin-2"-*O*-galactoside	0.263
612.169	$C_{27}H_{32}O_{16}$	[M+H]+	Okanin-4'-*O*-gentiobioside	0.536
624.168	$C_{28}H_{32}O_{16}$	[M+H]+	Nitensoside B	2.118
478.112	$C_{22}H_{22}O_{12}$	[M+H]+	Nepetin-7-*O*-glucoside(Nepitrin)	0.888
610.19	$C_{28}H_{34}O_{15}$	[M-H]-	Magnoloside E	0.367
594.158	$C_{27}H_{30}O_{15}$	[M+H]+	Luteolin-7-*O*-neohesperidoside (Lonicerin)	0.426
448.101	$C_{21}H_{20}O_{11}$	[M+H]+	Luteolin-4'-*O*-glucoside	3.173
448.101	$C_{21}H_{20}O_{11}$	[M+H]+	Luteolin-3'-*O*-glucoside	3.239
448.101	$C_{21}H_{20}O_{11}$	[M-H]-	Kaempferol-7-*O*-glucoside	0.931
814.216	$C_{35}H_{42}O_{22}$	[M+H]+	kaempferol-3-*O*-β-D-(2-*O* -6-acetylglucosyI) glucopyranoside-7- *O*-glucopyranoside	0.106

580.142	$C_{26}H_{28}O_{15}$	[M+H]+	Kaempferol-3-*O*-sambubioside	0.176
594.158	$C_{27}H_{30}O_{15}$	[M+H]+	Kaempferol-3-*O*-neohesperidoside	0.441
448.101	$C_{21}H_{20}O_{11}$	[M+H]+	Kaempferol-3-*O*-glucoside (Astragalin)	3.096
594.158	$C_{27}H_{30}O_{15}$	[M+H]+	Kaempferol-3-*O*-glucorhamnoside	0.478
448.101	$C_{21}H_{20}O_{11}$	[M-H]-	Kaempferol-3-*O*-galactoside (Trifolin)	0.924
534.101	$C_{24}H_{22}O_{14}$	[M+H]+	Kaempferol-3-*O*-(6"-malonyl)glucoside	0.499
534.101	$C_{24}H_{22}O_{14}$	[M+H]+	Kaempferol-3-*O*-(6"-malonyl)galactoside	0.494
773.213	$C_{33}H_{41}O_{21}$	[M+H]+	kaempferol-3-*O*-(2-*O*-glucosyl)glucoside-7-O-glucoside	1.213
490.111	$C_{23}H_{22}O_{12}$	[M+H]+	Kaempferol-3-*O*-(2"-acetyl)glucoside	1.753
610.153	$C_{27}H_{30}O_{16}$	[M+H]+	Kaempferol-3,7-di-O-glucoside	0.831
490.111	$C_{23}H_{22}O_{12}$	[M+H]+	kaempferol 3-*O*-(6-*O*-acetyl) glucopyranoside	0.529
652.163	$C_{29}H_{32}O_{17}$	[M+H]+	kaempferol 3-*O*-(6-*O*-acety1) glucopyranoside-7-O-glucopyranoside	2.716
652.164	$C_{29}H_{32}O_{17}$	[M+H]+	kaempferol 3-*O*-(2-*O*-acetylglucosyl)glucoside	2.806
286.048	$C_{15}H_{10}O_{6}$	[M-H]-	Kaempferol (3,5,7,4'-Tetrahydroxyflavone)	0.419
478.111	$C_{22}H_{22}O_{12}$	[M+H]+	Isotamarixin	0.169
478.111	$C_{22}H_{22}O_{12}$	[M+H]+	Isorhamnetin-7-*O*-glucoside (Brassicin)	2.710
624.162	$C_{28}H_{32}O_{16}$	[M+H]+	Isorhamnetin-3-*O*-rutinoside (Narcissin)	1.977

624.169	$C_{28}H_{32}O_{16}$	[M+H]+	Isorhamnetin-3-*O*-glucoside-7-*O*-rhamnoside	2.118
478.111	$C_{22}H_{22}O_{12}$	[M+H]+	Isorhamnetin-3-*O*-Glucoside	2.491
448.1	$C_{21}H_{20}O_{11}$	[M+H]+	Isorhamnetin-3-*O*-arabinoside	0.369
624.169	$C_{28}H_{32}O_{16}$	[M+H]+	Isorhamnetin 4'-neohesperidoside	2.175
316.058	$C_{16}H_{12}O_7$	[M-H]-	Isorhamnetin	0.793
464.095	$C_{21}H_{20}O_{12}$	[M+H]+	Isohyperoside	1.395
194.058	$C_{10}H_{10}O_4$	[M-H]-	Isoferulic Acid	0.104
312.136	$C_{19}H_{20}O_4$	[M+H]+	Isobavachalcone D	0.202
464.132	$C_{22}H_{24}O_{11}$	[M-H]-	Hesperetin-5-*O*-glucoside	1.180
610.132	$C_{30}H_{26}O_{14}$	[M+H]+	helichrysoside	0.765
610.132	$C_{30}H_{26}O_{14}$	[M+H]+	Gallocatechin-(4α→8)-gallocatechin	0.732
198.053	$C_9H_{10}O_5$	[M-H]-	Gallic Acid Ethyl Ester; Ethyl gallate	0.320
170.022	$C_7H_6O_5$	[M-H]-	Gallic acid	0.108
610.19	$C_{28}H_{34}O_{15}$	[M+H]+	Forsythiaside J	0.147
286.048	$C_{15}H_{10}O_6$	[M+H]+	Fisetin	0.519
194.058	$C_{10}H_{10}O_4$	[M-H]-	Ferulic acid	0.102
166.063	$C_9H_{10}O_3$	[M-H]-	Ethylparaben	0.115

450.116	$C_{21}H_{22}O_{11}$	[M+H]+	Eriodictyol-7-*O*-glucoside	0.153
278.152	$C_{16}H_{22}O_{4}$	[M+H]+	Diisobutyl phthalate	1.636
278.152	$C_{16}H_{22}O_{4}$	[M+H]+	Dibutyl phthalate	1.991
789.209	$C_{33}H_{41}O_{22}$	[M+H]+	Delphinidin-3-*O*-sophoroside-5-*O*-glucoside	0.124
449.108	$C_{21}H_{21}O_{11}$	[M+H]+	Cyanidin-3-*O*-glucoside (Kuromanin)	0.782
449.108	$C_{21}H_{21}O_{11}$	[M+H]+	Cyanidin-3-*O*-galactoside	0.782
611.161	$C_{27}H_{31}O_{16}$	[M+H]+	Cyanidin-3,5-*O*-diglucoside (Cyanin)	0.404
448.101	$C_{21}H_{20}O_{11}$	[M-H]-	Carthamone	0.649
278.152	$C_{16}H_{22}O_{4}$	[M+H]+	Butyl isobutyl phthalate	2.180
594.137	$C_{30}H_{26}O_{13}$	[M+H]+	Biondnoid I	0.415
121	$C_{7}H_{7}NO$	[M+H]+	Benzamide	0.404
450.116	$C_{21}H_{22}O_{11}$	[M-H]-	Aromadendrin-7-*O*-glucoside	0.104
461.153	$C_{19}H_{27}NO_{12}$	[M-H]-	Anthranilate-1-*O*-Sophoroside	0.132
254.058	$C_{15}H_{10}O_{4}$	[M+H]+	7,8-Dihydroxy-4-phenylcoumarin	0.148
192.042	$C_{10}H_{8}O_{4}$	[M+H]+	7,8-Dihydroxy-4-methylcoumarin	0.231
478.111	$C_{22}H_{22}O_{12}$	[M+H]+	6-Methoxykaempferol-3-*O*-glucoside	0.658
626.148	$C_{27}H_{30}O_{17}$	[M+H]+	6'-Hydroxykaempferol-6,7-*O*-Diglucoside	0.197

626.148	$C_{27}H_{30}O_{17}$	[M+H]+	6'-Hydroxykaempferol-3,6-*O*-Diglucoside	0.211
478.111	$C_{22}H_{22}O_{12}$	[M+H]+	6-C-Methylquercetin-3-*O*-glucoside	0.577
450.116	$C_{21}H_{22}O_{11}$	[M-H]-	6-C-Glucosyl-2-Hydroxynaringenin	0.108
168.042	$C_8H_8O_4$	[M+H]+	5-Methoxysalicylic acid	0.177
192.042	$C_{10}H_8O_4$	[M+H]+	5,7-Dihydroxy-4-methylcoumarin	0.191
610.189	$C_{28}H_{34}O_{15}$	[M-H]-	4-*O*-(6'-*O*-Glucosylcaffeoylglucosyl)-4-hydroxybenzyl alcohol	0.409
139.027	$C_6H_5NO_3$	[M+H]+	4-Nitrophenol	0.639
300.085	$C_{13}H_{16}O_8$	[M-H]-	4-Hydroxybenzoyl glucose	0.187
122.037	$C_7H_6O_2$	[M-H]-	4-Hydroxybenzaldehyde	0.113
330.168	$C_{16}H_{26}O_7$	[M+H]+	4-hydroxy-2, 6, 6-trimethyl-1-cyclohexene-1-carbaldehyde-*O*-glucopyranoside	0.738
286.048	$C_{15}H_{10}O_6$	[M-H]-	3,5,7,2'-Tetrahydroxyflavone; Datiscetin	0.363
254.058	$C_{15}H_{10}O_4$	[M+H]+	3,4'-Dihydroxyflavone	0.146
166.063	$C_9H_{10}O_3$	[M-H]-	3-(4-Hydroxyphenyl)-propionic acid	0.296
166.063	$C_9H_{10}O_3$	[M-H]-	2-Hydroxy-3-phenylpropanoic acid	0.198
288.063	$C_{15}H_{12}O_6$	[M-H]-	2-Hydroxy-2,3-dihydrogenistein	0.216
624.169	$C_{28}H_{32}O_{16}$	[M+H]+	2'-Hydoxy,5-methoxyGenistein-*O*-rhamnosyl-glucoside	2.033

640.164	$C_{28}H_{32}O_{17}$	[M+H]+	2'-Hydoxy,5-methoxyGenistein-4',7-*O*-diglucoside	0.943
166.063	$C_9H_{10}O_3$	[M-H]-	2,6-Dimethoxybenzaldehyde	0.193
